# Determining an optimal clinical dose of elobixibat, a novel inhibitor of the ileal bile acid transporter, in Japanese patients with chronic constipation: a phase II, multicenter, double-blind, placebo-controlled randomized clinical trial

**DOI:** 10.1007/s00535-017-1383-5

**Published:** 2017-08-24

**Authors:** Atsushi Nakajima, Mitsunori Seki, Shinya Taniguchi

**Affiliations:** 1 Department of Gastroenterology and Hepatology, Yokohama City University, 3-9 Fuku-ura, Kanazawa-ku, Yokohama, 236-0004 Japan; 2 Clinical Development Department, EA Pharma Co., Ltd, Tokyo, Japan; 3 R&D Planning Department, EA Pharma Co., Ltd, Tokyo, Japan

**Keywords:** Elobixibat, Ileal bile acid transporter, Bile acids, Chronic constipation, Spontaneous bowel movement

## Abstract

**Background:**

Elobixibat is an oral treatment candidate for chronic constipation with a novel mechanism of action via inhibition of the ileal bile acid transporter. We performed this randomized, double-blind, placebo-controlled, dose-finding phase IIb study in Japanese patients with chronic constipation to determine the optimal clinical dose of elobixibat.

**Methods:**

Japanese patients with chronic constipation were randomized to receive elobixibat (5, 10, or 15 mg) or placebo once daily for 2 weeks. The primary efficacy endpoint was the change from baseline in frequency of spontaneous bowel movements at Week 1 of treatment. Secondary endpoints and adverse events were also examined.

**Results:**

Among 226 patients who provided informed consent, 163 patients were randomized and included in the full analysis set. In the 10- and 15-mg groups, frequency of spontaneous bowel movements (±standard deviation) were significantly higher than baseline (5.7 ± 4.2 and 5.6 ± 3.5 times per week, respectively, compared with 2.6 ± 2.9 times per week in the placebo group [*P* = 0.0005, *P* = 0.0001, respectively]). Subgroup analysis indicated that elobixibat was equally effective in patients with or without constipation-predominant irritable bowel syndrome. Common adverse events included mild abdominal pain and diarrhea in the elobixibat groups; no serious or severe adverse events occurred. Elobixibat was well tolerated at once-daily oral doses up to 15 mg for 2 weeks.

**Conclusions:**

Our study results suggest that 10 mg of elobixibat is a clinically optimal dose for Japanese patients with chronic constipation.

**Clinical trial registration number:**

JapicCTI-142608.

## Introduction

Chronic constipation is one of the most common chronic gastrointestinal conditions, generally characterized by decreased frequency and irregular intervals of bowel movements, changes in stool consistency, straining during bowel movements, and the sensation of incomplete evacuation. Chronic constipation is most often defined according to the Rome III diagnostic criteria of functional constipation published in 2006 [1]. (In the Rome IV diagnostic criteria released in 2016, there were no changes to the diagnostic criteria for functional constipation [2]). Chronic constipation reportedly affects 14–17% of the population [3, 4], occurring more frequently in females and the elderly [5]; it adversely affects both physical and psychological quality of life [6], while simultaneously impairing economic productivity [7]. In a survey of 557 patients with chronic constipation in the United States, about half of respondents were not satisfied with their treatment regimen due to concerns regarding efficacy and safety [8]. According to an online survey of 5155 Japanese subjects, 28.4% of respondents considered themselves to be constipated, revealing that chronic constipation is a ubiquitous problem in Japan [9]. Because chronic constipation arises from multiple causes, no single or combined treatment has been shown to be effective for all chronic constipation patients; thus, there is an unmet need for new, effective treatment options [10, 11].

Elobixibat is a novel ileal bile acid transporter inhibitor that is expressed in the terminal ileum for treatment of chronic constipation [12]. By inhibiting bile acid reabsorption, elobixibat increases the amount of bile acid reaching the large intestine, which subsequently enhances colonic motility and secretion [13]. Elobixibat acts locally in the gut, resulting in minimal systemic exposure [14]. It is well known that bile acids induce diarrhea, and when the enterohepatic circulation of bile acids is broken due to ileal disease or resection, excessive quantities of bile acids may enter the colon, thereby resulting in diarrhea [15, 16]. By exploiting this function of bile acids, elobixibat provides a potential new treatment for chronic constipation.

In the United States, a clinical study was conducted in patients with functional constipation to evaluate the effects of elobixibat on small intestinal and colonic transit. At a dose of 15 or 20 mg once daily for 14 days, elobixibat accelerated overall colonic transit with no effect on small intestinal transit [17]. A phase IIb study, also conducted in the United States, examined the therapeutic effect of elobixibat in patients with chronic idiopathic constipation (CIC). There was a clinically and statistically significant dose–response relationship observed for once-daily doses of elobixibat at 5, 10, or 15 mg. Elobixibat increased stool frequency and improved constipation-related symptoms, e.g., straining and distention. Effects were maintained over 8 weeks of treatment [18].

This article reports the results of a phase IIb dose–response clinical study and determination of the optimal clinical dose of elobixibat in Japanese patients with chronic constipation.

## Methods

### Study design and procedures

A multicenter, randomized, double-blind, placebo-controlled, parallel-group, phase IIb study was conducted between July and December 2014 (from the first informed consent to the last patient observation) at 16 sites in Japan. The study was registered in the clinical study database of the Japan Pharmaceutical Information Center (JapicCTI-142608).

After providing informed consent, patients underwent vital sign measurement and laboratory testing to confirm eligibility for participation in the study. Patients were provisionally enrolled 16 days prior to the planned day of treatment initiation. Patients then entered a 2-week screening period, during which eligibility was confirmed based on bowel movements occurring from Day −15 to Day −2. If organic constipation had not been previously ruled out by colonoscopy or barium enema within the past 5 years, colonoscopy was performed at least 8 days before the start of the screening period to exclude the possibility of organic conditions. Based on a predetermined randomization table by the permuted block method with 8 patients per group, the patient enrollment center randomized eligible patients to one of four groups: 5-, 10-, and 15-mg elobixibat, and placebo. Randomized patients received the study treatment once daily before breakfast for 14 days beginning on the day after randomization. Rescue medication (bisacodyl suppository, 10 mg) was allowed only for patients who experienced no bowel movement for at least 72 consecutive hours between the start of the screening period and the last observation.

To evaluate efficacy, patient diaries were used to investigate the date and time of bowel movements, assessment of stool consistency on a scale from 1 (hard lumps) to 7 (liquid consistency) according to the Bristol Stool Form Scale (BSFS), sensation of incomplete evacuation, and severity of constipation (assessed weekly). The investigational medical product was blinded by using a placebo tablet that was indistinguishable from the elobixibat 5-mg tablet in terms of appearance, odor, and volume. The randomization table was appropriately retained to ensure the blindness of the study.

### Study population

This study included male and female outpatients 20–74 years of age who satisfied the Rome III diagnostic criteria for functional constipation, which excludes rectoanal abnormalities. This study included patients with constipation-predominant irritable bowel syndrome (IBS-C), although the Rome III diagnostic criteria specify that patients should be diagnosed with functional constipation only when they do not meet the diagnostic criteria for IBS-C.

All patients in the study met the inclusion criteria of spontaneous bowel movements (SBMs) occurring fewer than 3 times per week for at least 6 months, with fewer than 6 SBMs during the 2-week screening period, in addition to one or more of the following symptoms associated with at least 25% of SBMs for at least 6 months: straining, lumpy or hard stools, and sensation of incomplete evacuation. In addition, inclusion criteria required the absence of organic lesions in the large intestine. All patients included in the study provided written informed consent. The study excluded patients who had (or were suspected to have) organic constipation, drug-induced constipation, or constipation induced by disease, such as hypothyroidism or Parkinson’s disease.

### Endpoints

The primary endpoint was the change in frequency of SBMs at Week 1 of treatment compared to Week 2 of the screening period (hereafter referred to as ‘baseline’). Secondary efficacy endpoints included the following six parameters: (1) change from baseline in the weekly frequency of SBMs at Week 2 of treatment; (2) change from baseline in the weekly frequency of complete SBMs (CSBMs), with CSBM defined as an SBM that is associated with a feeling of complete bowel emptying; (3) percentage of patients who experienced an initial SBM within 24 or 48 h of treatment; (4) time to first SBM; (5) stool consistency, as measured by BSFS; and (6) weekly severity of constipation evaluation. Safety endpoints were adverse events, laboratory tests, and vital signs.

### Ethical and legal aspects

This study was performed in accordance with the ethical principles established in the Declaration of Helsinki and Good Clinical Practice guidelines. In addition, the study protocol and informed consent form were approved by the central institutional review board (Yokohama Minoru Clinic and Kayaba Dermatology Clinic). All patients gave written informed consent before study participation. The study protocol and written information for informed consent were approved by institutional review boards. Patient identification codes were used to enroll and identify patients. Adequate consideration was given to protection of patients’ privacy.

### Sample size design

Based on prior phase II clinical studies in patients with CIC in the United States and a phase I study in Japanese patients with chronic constipation, we calculated the number of patients needed to detect significant differences at a significance level of 0.05 (two-sided) on the assumption that population changes from baseline in the frequency of SBMs were 1.52 times in the placebo group and 3.59 times in the elobixibat 10-mg group [18]. We determined that 34 patients per group were required at a statistical power of 80%, and 44 patients per group were required at a power of 90%. Accounting for treatment discontinuation, we set a target sample size of 44 patients per group. Patients were randomized to the treatment groups in a 1:1:1:1 ratio.

### Statistical analysis

Statistical Analysis System (SAS) Professional Version 9.3 software (SAS Institute Inc., Cary, NC, USA) was used for all statistical analyses.

Efficacy analysis was based on the full analysis set, defined as the population of all patients who were treated with the study drug at least once and had efficacy data. The safety analysis was based on the safety analysis set, which was defined as the population of all patients who were treated with the study drug at least once.

Throughout statistical analysis, we considered the issue of multiplicity of data, as the placebo group (control) was repeatedly compared with individual elobixibat groups. Specifically, analysis of covariance was performed by the closed testing procedure in which the placebo group was sequentially compared with elobixibat groups (15-, 10-, and 5-mg groups), with the frequency of SBMs at baseline as a covariate. Statistical testing was terminated when no significant differences were observed.

Frequency data were regarded as “missing” if frequencies of SBMs and/or CSBMs were evaluated fewer than 5 days in any week. Bowel movements within 24 h after the use of rescue medication were not regarded as spontaneous, and were considered unevaluable.

For BSFS, we calculated the mean weekly BSFS per patient. An analysis of covariance (ANCOVA) was conducted to assess changes in the elobixibat group versus the placebo group from baseline during the study treatment period, with mean weekly BSFS at baseline as a covariate.

Elobixibat, based on its mechanism of action of inhibiting the reabsorption of bile acids in the ileum, may decrease blood LDL-cholesterol. Post-treatment LDL cholesterol and HDL cholesterol levels were therefore disclosed after key code break to persons who were involved in this study and the study sponsor.

## Results

**Fig. 1:**
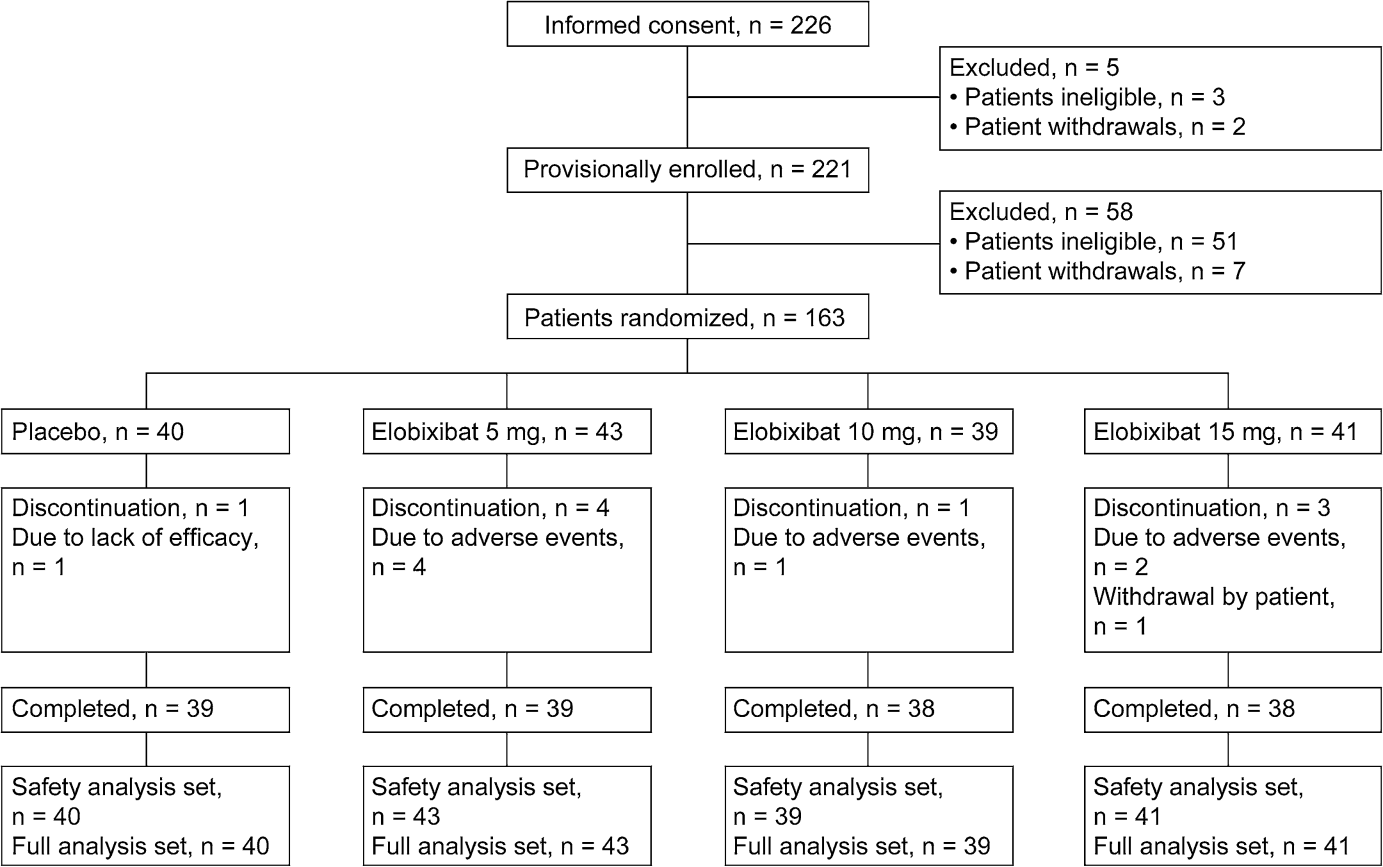
Disposition of patients

We planned to enroll a total of 176 patients, and assuming a drop-out rate of 20%, we received informed consent from a total of 226 patients. However, the drop-out rate was higher than originally estimated and, as a result, only 163 patients were enrolled. More importantly, only five patients discontinued in less than 5 days after treatment initiation, and these patients were excluded from the primary analysis. Therefore, we concluded that we could ensure sufficient statistical power of 80% or higher for primary endpoint achievement, and the patient enrollment was completed at 163 patients. Patients were randomized to placebo (40 patients), elobixibat 5-mg (43 patients), 10-mg (39 patients), and 15-mg (41 patients) groups. Treatment was completed in 39 patients each in the placebo and 5-mg groups, and in 38 patients each in the 10-mg and 15-mg groups. Reasons for treatment discontinuation were adverse events (7 patients), lack of efficacy (1 patient), and patient convenience (1 patient) (Fig. 1). Among 163 patients in the full analysis set, data for the frequency of SBMs at Week 1 of treatment were missing in 5 patients who discontinued the study drug before Day 5 (2 patients in the 5-mg group, 1 in the 10-mg group, and 2 in the 15-mg group).

There were no considerable differences in the demographics and baseline characteristics of patients with respect to sex, age, BMI, or the presence of IBS-C among the four groups. At baseline, the mean frequency of SBMs was 1.6–1.8 times per week in all groups (Table 1).

**Table 1:** Patient demographics and baseline characteristics

Characteristics	Placebo, *n* (%) *N* = 40	Elobixibat, *n* (%)		
		5 mg *N* = 43	10 mg *N* = 39	15 mg *N* = 41
Sex				
Male	2 (5.0)	5 (11.6)	4 (10.3)	9 (22.0)
Female	38 (95.0)	38 (88.4)	35 (89.7)	32 (78.0)
Age (years)^a^				
Mean ± SD	44.9 ± 12.2	46.1 ± 11.7	43.4 ± 13.4	43.9 ± 14.3
Min–max	21–72	25–73	20–66	20–73
BMI (kg/m^2^)				
Mean ± SD	21.40 ± 2.42	21.61 ± 2.73	21.93 ± 4.01	21.69 ± 3.63
Min–max	18.0–29.0	17.4–29.7	17.0–32.7	17.1–35.4
Constipation-predominant IBS				
No	28 (70.0)	26 (60.5)	29 (74.4)	30 (73.2)
Yes	12 (30.0)	17 (39.5)	10 (25.6)	11 (26.8)
The number of spontaneous bowel movements^b^ before receiving the study drug				
Mean ± SD	1.8 ± 1.1	1.8 ± 0.8	1.6 ± 1.0	1.8 ± 0.8
The number of complete spontaneous bowel movements^b^ before receiving the study drug				
Mean ± SD	0.8 ± 1.0	0.7 ± 0.9	0.6 ± 0.8	0.3 ± 0.6
Stool consistency measured by Bristol Stool Form Scale^b^				
Score 1–2	24 (60.0)	27 (62.8)	23 (59.0)	20 (48.8)
Score 3–5	9 (22.5)	13 (30.2)	10 (25.6)	19 (46.3)
Score 6–7	0 (0.0)	0 (0.0)	0 (0.0)	0 (0.0)

*BMI* body mass index, *IBS* irritable bowel syndrome, *SD* standard deviation

^a^Age is based on informed consent date of Visit 1

^b^Baseline value is based on Week 2 of the screening period

**Fig. 2:**
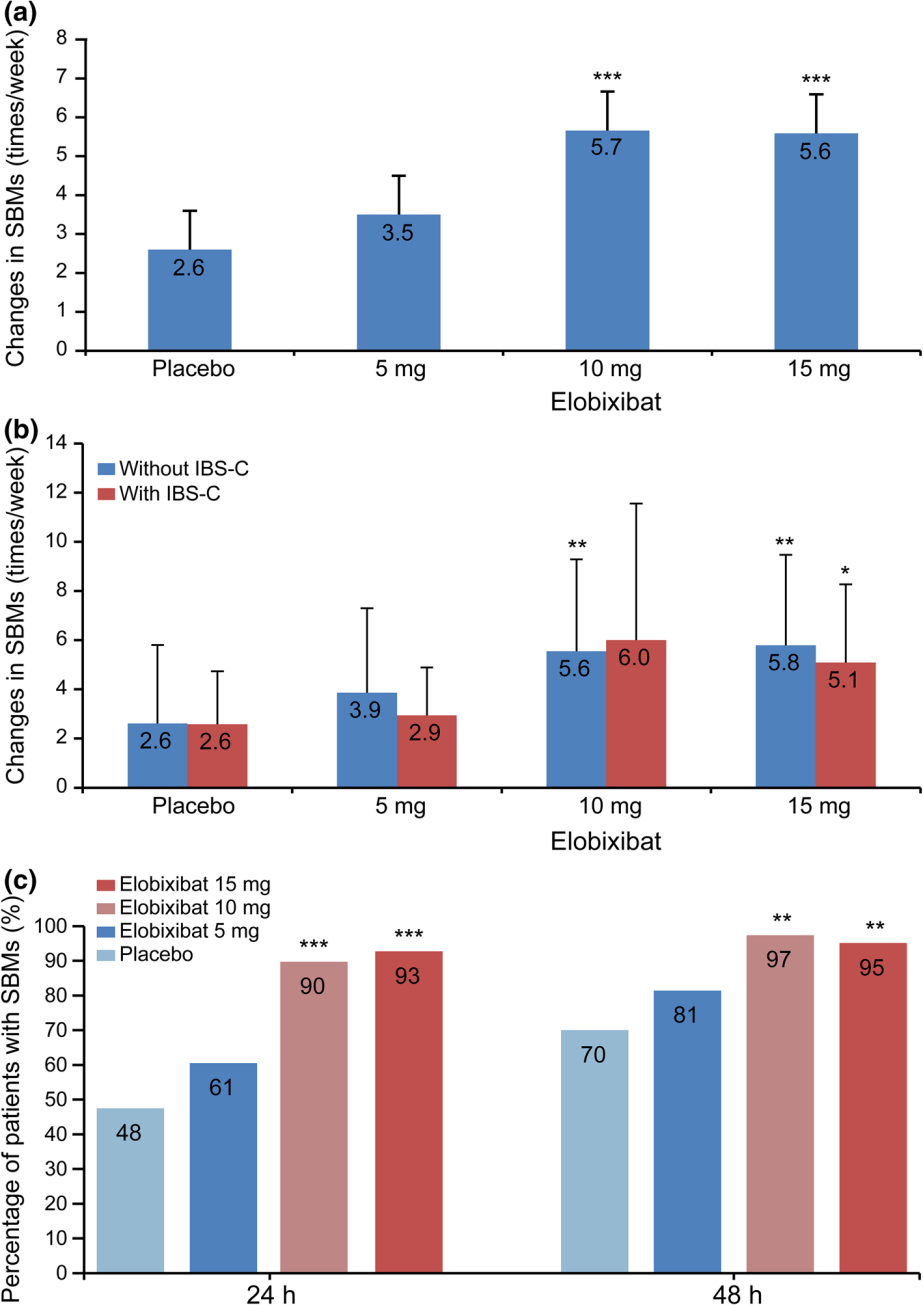
**a** Primary endpoint: change from baseline in SBMs at Week 1. Data are expressed as mean ± SD. ***P < 0.001 vs placebo (ANCOVA). **b** Evaluation of primary endpoint in patients with and without IBS-C. Data are expressed as mean ± SD. *P < 0.05, **P < 0.01 vs placebo (ANCOVA). The number of patients with IBS-C in the placebo and elobixibat 5, 10, and 15 mg groups was 12, 16, 9, and 11, respectively, and the number of patients without IBS-C in these groups was 28, 25, 29, and 28, respectively. *ANCOVA* analysis of covariance, *IBS*-C constipation-predominant irritable bowel syndrome, *SBM* spontaneous bowel movement, *SD* standard deviation. **c** Percentage of patients experiencing first SBM within 24 or 48 h after treatment initiation. Data are expressed as percentage. **P < 0.01, ***P < 0.001 vs placebo (Fisher’s Exact Test)

The frequency of SBMs at Week 1 (primary endpoint) significantly increased in the 10- and 15-mg elobixibat groups compared with the placebo group (*P* = 0.0005 and *P* = 0.0001, respectively; analysis of covariance). These results demonstrated a dose-dependent increase in SBMs from the 5- to 10-mg dose, but similar SBM values between the 10- and 15-mg doses (Fig. 2a). Results from Week 2 showed significant increases in the frequency of SBMs in all elobixibat groups compared with the placebo group (data not shown). A subgroup analysis examined the change from baseline in the primary endpoint in patients with or without IBS-C. Frequency of SBMs significantly increased in the 10- and 15-mg groups without IBS-C and in the 15-mg group with IBS-C, compared with the placebo group (Fig. 2b). The percentages of patients who experienced the first SBM within 24 or 48 h after treatment initiation were significantly higher in the 10- and 15-mg groups compared with the placebo group (Fig. 2c). The mean time to first SBM for each group was as follows (median time to first SBM): placebo, 36.2 h (24.3 h); 5-mg elobixibat group, 19.9 h (5.8 h); 10-mg group, 8.2 h (4.8 h); and 15-mg group, 8.5 h (3.5 h). These results showed dose-dependent increases up to the 10-mg dose, with similar values between the 10- and 15-mg groups.

**Fig. 3:**
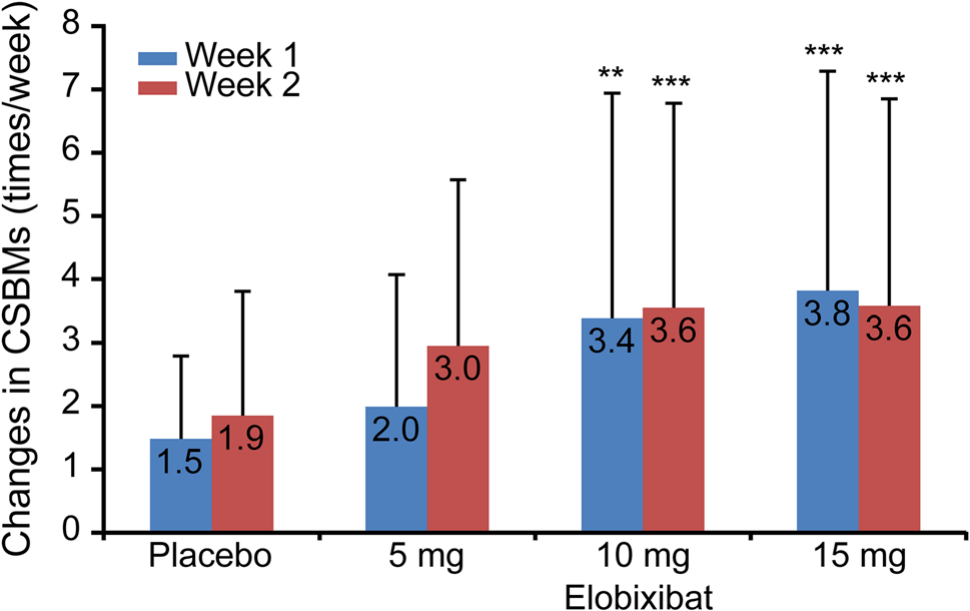
Weekly change from baseline in frequency of CSBMs. Data are expressed as mean ± SD. **P < 0.01, ***P < 0.001 vs placebo (ANCOVA). *CSBM* complete spontaneous bowel movement, *ANCOVA* analysis of covariance, *SD* standard deviation

Our results show significantly larger changes in the frequency of CSBMs in the 10- and 15-mg elobixibat groups compared with the placebo group throughout the study period (Week 1 of treatment: 10-mg group, *P* = 0.0032, and 15-mg group, *P* = 0.0002; Week 2 of treatment: 10-mg group, *P* = 0.0004, and 15-mg group, *P* = 0.0007) (Fig. 3).

**Fig. 4:**
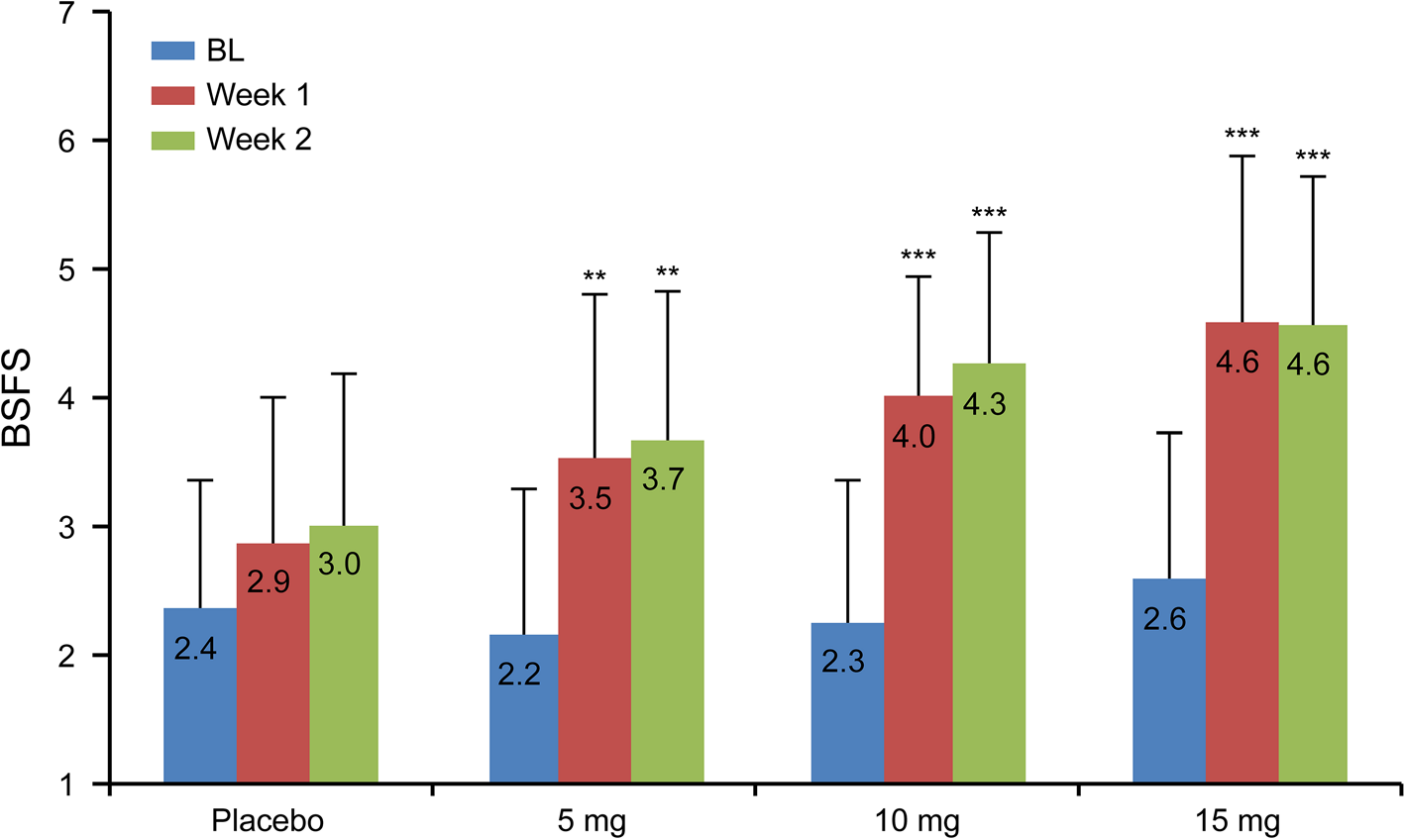
Stool consistency measured by BSFS. Data are expressed as mean ± SD. **P < 0.01, ***P < 0.001 (ANCOVA). *ANCOVA* analysis of covariance, *BL* baseline, *BSFS* Bristol Stool Form Scale, *SD* standard deviation

**Fig. 5:**
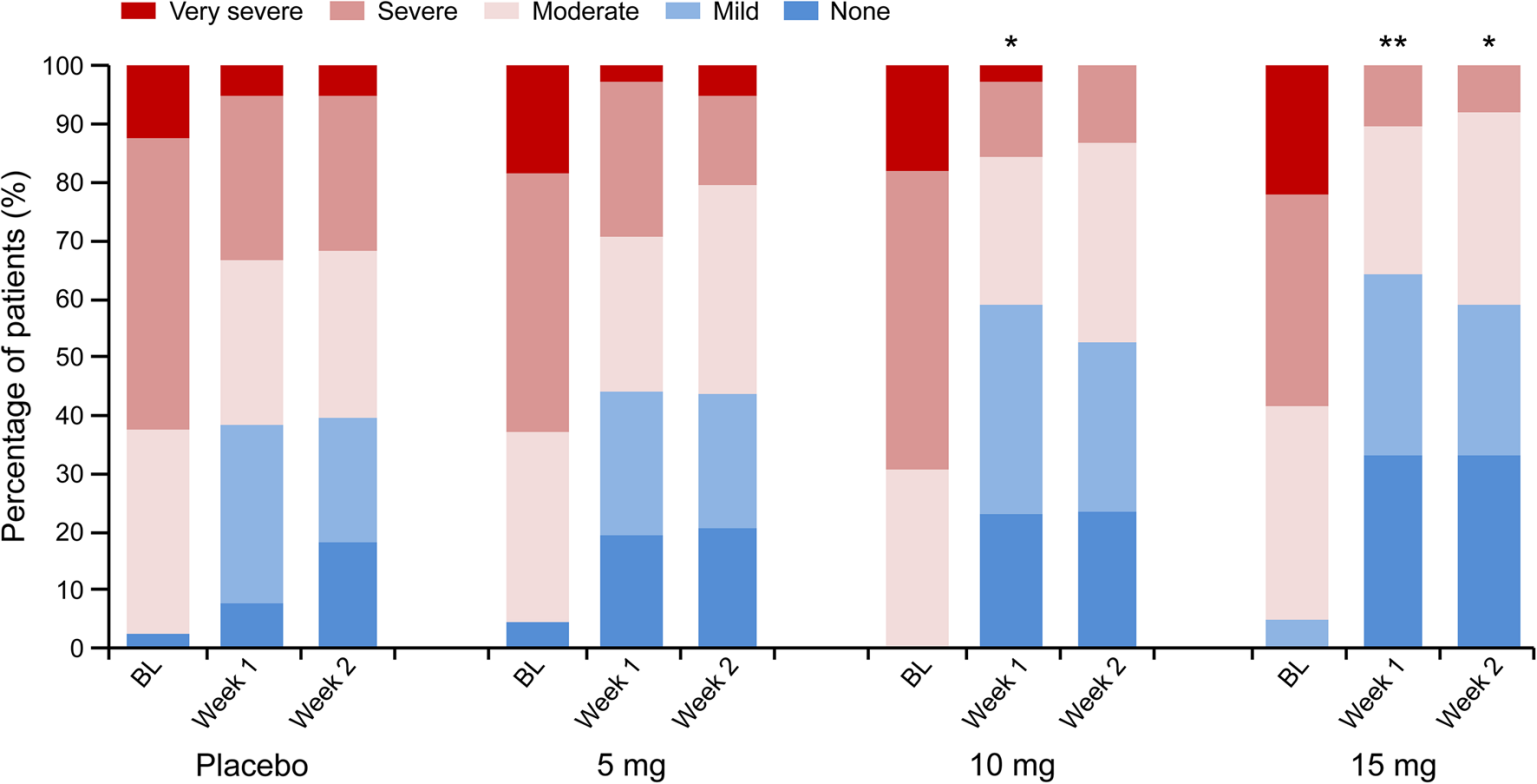
Evaluation of weekly severity of constipation. Data are expressed as mean ± SD. *P < 0.05, **P < 0.01 vs placebo (Wilcoxon rank sum test). Constipation severity score: none, mild, moderate, severe, very severe. *BL* baseline, *SD* standard deviation

BSFS was close to the ideal stool consistency of “4” in the 10-mg group (Fig. 4). Weekly constipation severity scores were significantly improved in the 10- and 15-mg groups at Week 1 of treatment (*P* = 0.0224, *P* = 0.0018), as well as the 15-mg group at Week 2 of treatment (*P* = 0.0172), compared with the placebo group (Fig. 5).

### Safety

The incidence of adverse events was higher in all elobixibat groups compared with the placebo group. However, no adverse events were serious or severe. Treatment was discontinued due to adverse events in 4 patients in the 5-mg group, 1 patient in the 10-mg group, and 2 patients in the 15-mg group. Adverse events leading to discontinuation were “diarrhea and abdominal pain”, “diarrhea, abdominal pain, and nausea”, “defecation urgency and abdominal pain”, “dizziness, feeling abnormal, yawning, and loss of consciousness” in 1 patient each in the 5-mg group; “headache, nausea, lower abdominal pain, and malaise” in 1 patient in the 10-mg group; and “diarrhea and abdominal pain” in 2 patients in the 15-mg group. The most common adverse events were gastrointestinal disorders including abdominal pain and diarrhea; most events were mild and of no clinical significance (Table 2).

**Table 2:** Discontinuation and study-related adverse events in the gastrointestinal tract

	Placebo, *n* (%) *N* = 40	Elobixibat, *n* (%)		
		5 mg *N* = 43	10 mg *N* = 39	15 mg *N* = 41
Any adverse events	6 (15.0)	18 (41.9)	12 (30.8)	8 (19.5)
Study-related adverse events	2 (5.0)	14 (32.6)	11 (28.2)	7 (17.1)
Adverse events leading to discontinuation	0 (0.0)	4 (9.3)	1 (2.6)	2 (4.9)
Gastrointestinal disorders				
Total	2 (5.0)	12 (27.9)	11 (28.2)	6 (14.6)
Mild	2 (5.0)	12 (27.9)	10 (25.6)	4 (9.8)
Moderate	0 (0.0)	0 (0.0)	1 (2.6)	2 (4.9)
Severe	0 (0.0)	0 (0.0)	0 (0.0)	0 (0.0)
Abdominal pain	0 (0.0)	10 (23.3)	10 (25.6)	5 (12.2)
Diarrhea	0 (0.0)	4 (9.3)	2 (5.1)	3 (7.3)
Abdominal distension	0 (0.0)	3 (7.0)	0 (0.0)	1 (2.4)
Abdominal pain, lower	1 (2.5)	0 (0.0)	1 (2.6)	1 (2.4)
Abdominal pain, upper	1 (2.5)	0 (0.0)	1 (2.6)	0 (0.0)
Defecation urgency	0 (0.0)	1 (2.3)	0 (0.0)	0 (0.0)
Nausea	0 (0.0)	1 (2.3)	1 (2.6)	0 (0.0)
Vomiting	0 (0.0)	0 (0.0)	1 (2.6)	0 (0.0)

There were no clinically significant changes in laboratory tests or vital signs between groups, with the exception of LDL cholesterol. Compared with the placebo group, the 5-, 10-, and 15-mg elobixibat groups showed significant decreases in LDL cholesterol from baseline levels after 14 days of treatment (*t* test; *P* = 0.0118, *P* < 0.001, *P* = 0.0067, respectively). The mean ± standard deviation actual LDL cholesterol level (mg/dL) before and after administration of elobixibat (mean change from baseline ± standard deviation) was 117.6 ± 32.3 and 119.2 ± 30.6 (1.6 ± 13.1), respectively, in the placebo group; 110.1 ± 23.9 and 103.7 ± 21.7 (−6.4 ± 14.9), respectively, in the 5-mg elobixibat group; 119.0 ± 35.1 and 105.1 ± 32.1 (−13.8 ± 14.8), respectively, in the 10-mg group; and 111.7 ± 26.3 and 104.1 ± 23.9 (−7.8 ± 16.9), respectively, in the 15-mg group. There were no significant changes in HDL cholesterol in all groups.

## Discussion

Bile acids promote colonic secretion through the physiological mechanisms of intracellular activation of adenylate cyclase, increased mucosal permeability, and inhibition of apical Cl^−^/OH^−^ exchange [19–22]. Bile acids also induce propulsive contractions in the mammalian and human colon [23]. Moreover, bile acids activate colonic motility and secretion in humans [24, 25]. Based on this evidence, elobixibat, with a novel mechanism of action that inhibits reabsorption of bile acids, is anticipated as a new candidate for the treatment of constipation.

In this study of 163 Japanese patients with chronic constipation, elobixibat, at doses of 10 and 15 mg, was associated with a significant change from baseline in the frequency of SBMs at Week 1 of treatment (primary endpoint) compared with placebo. Elobixibat also significantly outperformed placebo in terms of most secondary endpoints, such as the frequency of CSBMs and stool consistency by BSFS. These results show that elobixibat, in once-daily oral doses of 10 and 15 mg, benefits patients with chronic constipation. Stool consistency and constipation severity scores were improved to a greater extent in the 15-mg group compared with the 10-mg group, but there was not a significant difference between the two groups. Although elobixibat also showed benefits at a dose of 5 mg, these effects were inferior to those associated with the 10- and 15-mg doses. Based on these findings, the recommended once-daily oral dose of elobixibat was 10 mg.

Elobixibat was well tolerated in patients with chronic constipation at once-daily oral doses up to 15 mg. The most common adverse events were gastrointestinal disorders such as abdominal pain and diarrhea. Most of these symptoms were mild with very few cases of moderate symptoms. With regards to abdominal pain specifically, it has been reported that certain bile acids are prokinetic in the colon, stimulating propagated contractions [13, 24]. Compared with healthy controls, patients with constipation showed fewer pressure waves and a lower incidence of propagated contractions [26]. As these propagated contractions can be painful, the abdominal pain associated with elobixibat observed in our study was likely related to these propagated contractions stimulated by increased bile acids in colon [27].

A subgroup analysis of primary endpoint data based on the absence or presence of IBS-C showed similar results regardless of IBS-C status. Furthermore, subgroup analysis of patients stratified by IBS-C status showed that there was no significant difference in the frequency of adverse events between patients with IBS-C and patients without IBS-C (Table 3). These data suggest that elobixibat is effective for patients with or without IBS-C. To our knowledge, this is the first report of the effectiveness of elobixibat in improving SBMs in Japanese patients with IBS-C; however, further research is necessary, as IBS symptom relief was not addressed in our current study.

**Table 3:** Subgroup analysis for study-related adverse events

Constipation-predominant IBS (IBS-C)	Placebo, *n* (%) *N* = 40	Elobixibat, *n* (%)		
		5 mg *N* = 43	10 mg *N* = 39	15 mg *N* = 41
Without IBS-C				
Number of patients	28	26	29	30
Number of patients with an adverse event	2	9	9	7
Proportion of patients (%)	7.1	34.6	31.0	23.3
95% CI	1.98–22.65	19.41–53.78	17.28–49.23	11.79–40.93
With IBS-C				
Number of patients	12	17	10	11
Number of patients with an adverse event	0	5	2	0
Proportion of patients (%)	0.0	29.4	20.0	0.0
95% CI	0.00–24.25	13.28–53.13	5.67–50.98	0.00–25.88

*CI* confidence interval, *IBS* irritable bowel syndrome

The efficacy and safety of elobixibat were previously examined in a phase IIb clinical study in patients with CIC in the United States [18]. The study showed similar dose–response relationships with regard to the primary endpoint, while our present study showed a slightly higher change from baseline in SBMs in the placebo group and 10-mg group. The incidence of study-related gastrointestinal adverse events was essentially similar between the two studies. In both studies, abdominal pain was the most common adverse event, followed by diarrhea and abdominal distention. These findings suggest that there are no substantial ethnic differences in the efficacy or safety of elobixibat.

It has been previously reported that constipation is a risk factor for cardiovascular disease events [28]. In this trial, elobixibat elicited a decrease in LDL cholesterol levels with no effect on HDL cholesterol as a result of its mechanism of action; these findings were similar to those of the US study [17]. This beneficial effect on the lipid profile is a unique feature of elobixibat; improvement in LDL cholesterol levels might provide incremental benefits to a subset of chronic constipation patients also affected by dyslipidemia [29].

In conclusion, the results of this phase IIb study in Japanese patients with chronic constipation demonstrated that elobixibat significantly improved stool frequency and consistency, and was well tolerated up to 15 mg. Our study results suggest that 10 mg is a clinically optimal dose of elobixibat for Japanese patients with chronic constipation.
